# Ten years for PhysChem Forum-Japan (PCF-J)

**DOI:** 10.5599/admet.1452

**Published:** 2022-08-16

**Authors:** Takashi Mano

**Affiliations:** Ono Pharmaceutical Co.,Ltd.,1-1, Sakurai 3-chome, Shimamoto-cho, Mishima-gun, Osaka 618-8585, JAPAN, E-mail: mano@ono.co.jp; Tel.: +81-75-961-1151; Fax: +81-75-962-9314

**Keywords:** Physical-chemical property, oral absorption, PhysChem Forum, International Association of Physical Chemists, Konstantin Tsinman

## Abstract

The ten years of PhysChem Forum-Japan and Konstantin Tsinman’s great contributions to the forum are briefly described.

In 2012 several scientists of pharmaceutical physical-chemical properties working at Japanese companies got together and discussed how to improve recognition of the criticality of physicochemical properties in drug discovery and development from pharmaceutical science viewpoints. This was due to concerns that in industry, the assessment and application of physical-chemical properties of active pharmaceutical ingredients (APIs) such as solubility, lipophilicity, p*K*_a_’s, solubility product (*K*_sp_) and crystallinity might not be implemented properly in drug discovery and development processes, even though it was well recognised that the properties of APIs play important roles in oral drug absorption (*i.e.*, dissolution, permeation and even precipitation). If this is the case, it may lead to poor productivity in drug discovery and development.

Before starting PCF-J, some scientists had participated in the PhysChem Forum meetings in Europe and the International Association of Physical Chemists (IAPC) meetings. They acknowledged its values, for example, in cutting-edge and multidisciplinary topics, energetic scientific discussion by diverse participants from many countries and friendly networking [1,2]. Considering the practices of PhysChem Forum, they decided to set up a forum for pharmaceutical physical-chemical scientists in Japan. The original committee members organised the 1^st^ PhysChem Forum Japan (PCF-J) meeting on 25^th^ Feb. 2014 (a permission of the use of “PhysChem Forum” was granted by the ‘European’ PhysChem Forum in advance). PCF-J meetings were then held on an annual basis. Even during the COVID-19 pandemic, the meetings were successfully held online in 2020 and 2021, with more than one hundred participants from industry, academia (faculties and students) and regulatory bodies (Table 1).

The lead committee member of each PCF-J meeting upheld each theme, which tended to be dependent on the interests of the leader, and the PCF-J committee members constructed the programs. Applying the best practice of the IAPC meetings, the committee organized the Growth Project and invited several oversea lectures for the 4^th^ PCF-J meeting. Dr. Konstantin Tsinman was one of the lecturers that accepted our offer and volunteered to give lectures [3]. Konstantin gave an excellent talk on permeability, explaining the basics and applications of the theories of drug permeation in the gastrointestinal tract, including the unstirred water layer over the mucus layer. His talk extended to using the Parallel Artificial Membrane Permeability Assay (PAMPA) system and its application to improve the accuracy of permeability assessment. Konstantin included quizzes within his talk to arouse the audience’s curiosity, which created a harmonious atmosphere in the Growth Project session.

The contribution of Konstantin and other speakers from countries outside of Japan created the international environment in PCF-J, and the 6^th^ meeting was held as a joint meeting with IAPC as “Joint 7^th^ IAPC & 6^th^ PCF-J Meeting” in 2018 in Osaka, Japan [4]. This year (2022), the PCF-J 10^th^ meeting is scheduled for December 8^th^ and is planned to be held online. The committee is expecting as many as 100 participants.^5^

PCF-J has offered opportunities where pharmaceutical physical chemists meet in a multidisciplinary manner, and at the same time, new sciences and technologies meet to create new spaces for advancing theories and developing new methodologies. PCF-J has also helped many scientists grow. In its course, Konstantin’s significant contributions should not be forgotten. Also, we cannot forget his friendly and warm personality. We are so lucky that Konstantin’s paths and ours crossed and that we could share those moments with him. The committee members of PhysChem Forum-Japan continue to hold the Forum meetings, in one which Konstantin’s attendance was of great importance. His overall contributions greatly influenced all PCF-J meetings in the past and will continue in the future.

## Figures and Tables

**Table 1.** PhysChem Forum-Japan meetings.

No.	Date	Theme	Venue/Location
1	25^th^ Feb. 2014	Supersaturation	Hoshi Uni./Tokyo
2	11^th^ Dec. 2014	From physical chemical property parameters to dissolution	The Mishima Chamber of Commerce and Industry/Shizuoka
3	25^th^ Nov. 2015	API design: API optimisation in drug discovery	Ono Pharmaceutical/Osaka
4	9^th^ Dec. 2016	Log P world: drug discovery from pharmaceutical molecular property perspectives	Asahi Kasei/Tokyo
5	1^st^-2^nd^ Dec. 2017	PAT Practices	The Mishima Chamber of Commerce and Industry/Shizuoka
6	28^th^-30^th^ Aug. 2018	IAPC-7/PCFJ-6 Joint International Symposium	Ritsumei Uni./Osaka
7	12^th^-13^th^ Nov. 2019	Application of mechanical-based simulation in CMC/pharmaceutical sciences	Eisai/Ibaraki
8	4^th^ Dec. 2020	Application of crystallisation and crystal structure analysis in drug discovery	Online
9	9^th^-10^th^ Dec. 2021	Nano: Drug substance or DDS, this is the question	Online
10	8^th^ Dec. 2022	IVIVC	Online
